# Verification of a Lachesis Symptom

**Published:** 1889-06

**Authors:** J. E. Russell

**Affiliations:** Brooklyn, N. Y.


### VERIFICATION OF A LACHESIS SYMPTOM.

I contracted a slight cold in the head from inadvertent exposure of the left side to the draught from an open window.

The effect was a gradually increasing sensitiveness of the whole left side of the head, which resulted in the most exquisite pain on the slightest movement of the head, while to touch the head with the hand was impossible; the bare approach of the hand to the head seemed to develop pain in each individual hair.

I thought it about time to interfere and modify its severity, and for that purpose took a dose of Lachesis, Johnstone potency CM.

In not longer than forty minutes thereafter I rose from my desk, and on turning the head suddenly discovered that I experienced no pain. I then passed my hand through the hair and over the whole left side of the head without there being a particle of sensitiveness or any pain.

I will merely say in closing that I had taken nothing for it previously, thinking it would pass away as other slight attacks had done.

I have seen the properly selected potentized drug do rapid work before, but have seldom seen as quick a response in so acute an attack as I experienced.

Brooklyn, N. Y. J. E. Russell.

